# Supplementary material for: Single cell spatial transcriptomics links Wnt signaling disruption to extracellular matrix development in a cleft palate model
Source: Sci Rep. 2025 Aug 13;15:29639. doi: 10.1038/s41598-025-14807-1 (PMC12350917; doi:10.1038/s41598-025-14807-1)
Supplement: Supplementary file 1 — Supplementary Material 1 [file 41598_2025_14807_MOESM1_ESM.docx]

**SUPPLEMENTAL FILE**

**Figure S1. Xenium In Situ Spatial Analysis Clarifies Genetic Programs Underlying Palatogenesis from E12.5 to E13.5 in Normal Development.**

a) A-P domains of comparison established using H&E staining and 10X Genomics Xenium *In Situ*. P_R_ right palatal shelf, P_L_ left palatal shelf, T tongue, TB_Mand_ mandibular tooth bud, TB_Max_ maxillary tooth bud. b – d) Full size volcano plots of differential gene expression highlight statistically significant (P-value < 0.05, |LG_2_FC| > 1) upregulation (red) and downregulation (blue) of genes identified in transcriptomic comparison of the whole palatal shelves (a), nasal domains (b), and oral domains (c) from E12.5 and E13.5, including the genes not pictured in Figure 2. All spatial transcriptomic analyses derived from n = 4 palatal shelf biological replicates.

**Figure S2. The *Pax9*^-/-^ Cleft Palate Demonstrates Disrupted Spatiotemporal Genetic Programs Across Oral and Nasal Domains.**

a) Aberrancies in the morphological transition from proliferation to elongation in Pax9^-/-^ cleft palate visualized with H&E staining and 10X Genomics Xenium Explorer v3.0 on E12.5 and E13.5. P_R_ right palatal shelf, P_L_ left palatal shelf, T tongue, TB_Mand_ mandibular tooth bud, TB_Max_ maxillary tooth bud. b – d) Volcano plots of differential gene expression highlight statistically significant (P-value < 0.05, |LG2FC| > 1) upregulation (red) and downregulation (blue) of genes identified in transcriptomic comparison of the whole palatal shelves (B), nasal domains (C), and oral domains (D) from E12.5 to E13.5. All spatial transcriptomic analyses derived from n = 4 palatal shelf biological replicates

**Figure S3. RNAscope Multiplex Validates Quantitative Spatial Transcriptomic Xenium In Situ Spatial Gene Expression Analysis**

a – d) Homeobox genes, *Wnt* modulators and effectors, and genes encoding ECM proteins hybridized in situ to confirm spatial patterns of differentiation: *Alx1* and *Prrx1* (homeobox); *Rspo1*, *Wif1*, and *Sparc* (Wnt modulator); *Wnt5a* (Wnt effector); *Eln* and *Col1a1*(fibrous protein); *Bgn* (SLRP) in palatal shelves of E12.5 and E13.5 wildtype versus *Pax9*^-/-^ models. All spatial transcriptomic analyses derived from n = 4 palatal shelves per stage of development (E12.5 and E13.5) and genotype (wildtype and *Pax9^-/-^*).

**Figure S4. Statistical Pipelines for Xenium *In Situ* Differential Gene Expression Analysis**

a) Diagram of the statistical pipeline for calculation of p-values from Xenium *In Situ* transcript density outputs. First, raw data outputs were Log_2_(X+1) transformed. Next, variance was calculated using an F-test (α = 0.0). Equal variance was assumed in cases #DIV/0 error. For genes with equal variance (F-test p-value >=0.05), a Student’s T-Test was performed. Alternatively, a Welch’s T-Test was performed if variance was determined to be unequal (F-test p-value <0.05). Finally, the Benjamini-Hochberg procedure was deployed as a false discovery rate control. b) Diagram of the statistical pipeline for calculation of LG_2_FCs from Xenium *In Situ* transcript density outputs. First, raw data was used to calculate the average transcript density data for each comparison group. Then, the fold change was calculated using the function shown and transformed via Log_2_ transformation.
