# Supplementary material for: Single cell spatial transcriptomics links Wnt signaling disruption to extracellular matrix development in a cleft palate model
Source: Sci Rep. 2025 Aug 13;15:29639. doi: 10.1038/s41598-025-14807-1 (PMC12350917; doi:10.1038/s41598-025-14807-1)
Supplement: Supplementary file 2 — Supplementary Material 2 [file 41598_2025_14807_MOESM2_ESM.docx]

Key Resources Table

| REAGENT or RESOURCE | SOURCE | IDENTIFIER |
| --- | --- | --- |
| Antibodies | | |
| Opal 520 Reagent Pack | Akoya Biosciences | FP1487001KT |
| Opal 570 Reagent Pack | Akoya Biosciences | FP1488001KT |
| Opal 690 Reagent Pack | Akoya Biosciences | FP1497001KT |
| Opal Polaris 780 Reagent Pack | Akoya Biosciences | FP1501001KT |
| Chemicals, peptides, and recombinant proteins | | |
| ProLong Gold Antifade Mountant | Thermo Fisher Scientific | P36930 |
| PBS | Sigma | Cat# D8537 |
| DAPI | Invitrogen | Cat# D1306 |
| RNAscope Wash Buffer | ACDBio/Bio-Techne | Cat# 310091 |
| Critical commercial assays | | |
| Visium HD Spatial RNA-sequencing | 10X Genomics, Inc. | Cat# 1000676 |
| Xenium In Situ RNA Localization | 10X Genomics, Inc. | Cat# 1000672 |
| RNAscope Multiplex Fluorescent Reagent Kit v2 | ACDBio/Bio-Techne | Cat# 323100 |
| RNAscope 4-Plex Ancillary Kit | ACDBio/Bio-Techne | Cat#323120 |
| Deposited data | | |
| Spatial RNA-sequencing raw data | Gene Expression Omnibus (GEO) | GSE284271 |
| Experimental models: Organisms/strains | | |
| Mouse: C57BL/6J | Charles River | JAX: 000664; RRID: IMSR JAX 000664 |
| Mouse: *Pax9^null^* | Zhou et al. 2011^24^ | N/A |
| Software and algorithms | | |
| Xenium Explorer | 10X Genomics, Inc. | Github: <https://github.com/quentinblampey/spatialdata_xenium_explorer> |
| Seurat | Satija Lab | Github: <https://github.com/satijalab/seurat> |
| Spatial Gene Expression Analysis Pipeline | Ziyi Wang | <https://labonom.github.io/GSE284271/00.NIH_10xVisiumHD.html> |
